# Current strategies and priorities for diabetic footwear design and production: a cross-European exploratory survey of clinicians and shoemakers

**DOI:** 10.1007/s00592-026-02669-6

**Published:** 2026-02-18

**Authors:** Hadi Sarlak, Kamran Shakir, Giulia Rogati, Alberto Leardini, Lisa Berti, Paolo Caravaggi

**Affiliations:** 1 Department of Biomedical and Neuromotor Sciences, Alma Mater Studiorum - Università di Bologna, Via Zamboni,33, Bologna, 40126 Italy; 2 Movement Analysis Laboratory and Functional Evaluation of Prostheses, IRCCS Istituto Ortopedico Rizzoli, Via di Barbiano 1/10, Bologna, 40136 Italy; 3 Physical Medicine and Rehabilitation Unit, IRCCS Istituto Ortopedico Rizzoli, Bologna, 40136 Italy

**Keywords:** Diabetic foot ulcers, Therapeutic footwear, Offloading, Survey, Footwear design

## Abstract

**Background:**

Therapeutic footwear plays an important role in preventing ulceration in people with diabetes. Despite guidelines recommending offloading footwear for individuals at risk of ulceration, limited data are available on the alignment between current industrial practices, clinical expectations, and evidence. This study explored current practices, priorities and challenges associated with diabetic footwear from manufacturers’ and clinicians’ perspectives across Europe.

**Methods:**

An exploratory cross-sectional survey was conducted between May and October 2025 to gather insights from diabetic footwear manufacturers and clinicians involved in diabetic foot care across Europe. A 26-item questionnaire was developed to explore product design, materials, innovation, and adherence to guidelines among manufacturers. Additionally, a separate 14-item questionnaire for clinicians examined footwear prescription, patient barriers, and industry communication. Quantitative data were analysed descriptively, and open-text responses underwent thematic analysis.

**Results:**

Nine manufacturers and twelve clinicians participated in the survey. While only half of the Manufacturers that completed the survey reported having a research and development department, most reported adopting data-driven design approaches (*n* = 8) and scientific literature (*n* = 7). Offloading, internal volume, and toe protection were the highest-ranking priorities for manufacturers, whereas aesthetics ranked lowest. Clinicians, conversely, prioritised accommodation of deformities, offloading, and comfort, and highlighted poor aesthetics and shoe weight as major limitations to adherence.

**Conclusions:**

This exploratory study suggests partial alignment between diabetic footwear manufacturers and clinicians on functional and offloading features but highlights gaps in aesthetics, materials, and sustainability. Better collaboration, data-driven innovation, and clearer product specifications could improve user adherence, prescription efficacy, and the preventive role of therapeutic footwear in diabetes care.

**Supplementary Information:**

The online version contains supplementary material available at 10.1007/s00592-026-02669-6.

## Introduction

Diabetic foot ulcers (DFUs), one of the most severe complications of diabetes mellitus (DM), are associated with a high degree of morbidity and mortality, as well as substantially higher healthcare costs if not treated properly and in a timely manner [1, 2]. The areas of the plantar foot subjected to high peak pressure and repetitive stress, especially with peripheral neuropathy, are more likely to develop new or recurrent DFUs [1]. Offloading of at-risk foot regions is thus an essential part of DFU prevention and healing [3], and a longstanding preventive strategy recommended by clinicians to prevent ulceration in people with DM at risk of DFUs [4].

The International Working Group on the Diabetic Foot (IWGDF) recommends the use of accommodating offloading shoes with custom-made insoles for people at medium to high risk of DFUs [5]. In fact, adherence to therapeutic shoes has been shown to be associated with a decrease in recurrence rates [6–8]. However, the variability in study design, intervention type, control shoes, and patient adherence have led to inconsistent results, which hinders the provision of stronger recommendations in favour of therapeutic shoes [9, 10]. Data on the prevention of first ulceration using off-the-shelf diabetic shoes are almost non-existent [11]. However, since ulceration of high-risk plantar regions is significantly associated with plantar pressure [12], the offloading features of therapeutic shoes are often prioritised to devise the optimal design criteria for diabetic footwear.

Therapeutic footwear should be designed to reduce peak pressures, accommodate foot deformities, and provide stability and protection to at-risk individuals. However, as frequently reported, user adherence to these shoes is often low, possibly due to discomfort, unattractive appearance, high weight and low stability [13–15]. Low adherence to therapeutic footwear can undermine its protective properties [4], and as such, the design and perceived comfort play a critical role in the overall effectiveness of this intervention.

Traditionally, footwear manufacturing and personalisation have relied on the expertise and experience of the shoemaker. More recently, the design of therapeutic footwear can be informed by morphometric and functional data, such as 3D scans and baropodometric data [16, 17]. This data-driven approach to the development of diabetic footwear, which incorporates objective measurements and advanced technology to develop and optimise footwear, represents a substantial shift from traditional methods. While the extent to which this approach has been implemented across the therapeutic footwear industry remains unclear, there is potential to design footwear capable of significantly reducing DFU rates by improving fit and offloading properties [4, 18]. This potential is further boosted by novel additive manufacturing techniques, which are showing promising results in terms of prototyping and personalisation, and may be more sustainable than traditional mass-production strategies [19].

As far as the choice of the footwear materials, these have a significant effect on the foot/footwear mechanical interaction and on the overall lower-limb biomechanics by affecting shoe weight and mechanical properties such as the bending stiffness. While a substantial body of literature exists on insole materials and biomechanical properties [20, 21], shoe uppers and midsole/outsole components have a limited presence in the available literature [11].

The existing literature has mainly focused on isolated assessments of footwear and insoles, mainly with respect to the offloading properties. However, little is known about the incorporation of scientific literature and international guidelines into the development of diabetic footwear. Moreover, clinicians’ perspectives on the gaps in the current footwear market and on how well their expectations and experiences align with the diabetic footwear industry have not yet been explored.

To address these gaps, two separate questionnaires were developed and sent to a sample of diabetic footwear manufacturers and clinicians working in diabetic foot care across Europe. This study aimed to explore the challenges, opportunities, innovations, and areas of divergence in priorities between these two stakeholders.

## Materials and methods

Two distinct questionnaires were developed to explore the current perspective of diabetic footwear manufacturers and clinicians working in diabetic foot care (Table 1). The questions were designed by a panel consisting of 4 researchers in human biomechanics, a physician specialising in rehabilitation medicine with 20 years of experience, and an expert from the shoe manufacturing industry.

The first questionnaire comprised 26 multiple-choice ranking and open-ended questions, aimed at exploring the development and manufacturing process, market research and demand, innovation, user feedback, design, and materials of diabetic shoes, as well as compliance with international guidelines and scientific literature. A total of 44 European diabetic footwear manufacturers were identified through public repositories and search engines and invited by email to participate in this survey.

The second questionnaire, comprising 14 multiple-choice, ranking and open-ended questions, was sent to 16 clinicians working in diabetic foot care centres across Europe through professional networks and snowballing. This survey focused on footwear practices, including prescription and provision, patient feedback, challenges, expectations, and the perspectives of participating clinicians. The order of the options in the ranking questions was randomised for each participant in both questionnaires.

**Table 1:** Summary of the structure and methodological characteristics of the two questionnaires used in this study, detailing their target populations, thematic domains, data collection methods, and analytical approaches

Characteristics	Survey of Manufacturers	Survey of Clinicians
Target population	Representatives from European Manufacturers producing diabetic footwear	Clinicians (podiatrists, physicians, orthotists, prosthetists) working with diabetic foot patients across Europe
Study type	Cross-sectional observational survey	Cross-sectional observational survey
Main domains assessed	Company profile; product types; materials & design; guideline adherence; innovation & sustainability; feedback & R&D	Professional background; footwear provision; patient barriers; industry communication; development priorities
Data collection mode	Online self-administered, English (May–October 2025)	Online self-administered, English (May–October 2025)
Analytical methods	Quantitative data analysed descriptively; open-text responses analysed thematically to identify recurring insights (inductive).	Quantitative data analysed descriptively; qualitative responses were subjected to thematic analysis (inductive).
Number of items	26	14

### Data collection and analysis

The data collection timeframe was from May 2025 until October 2025. Quantitative data from multiple-choice and ranking questions were analysed using descriptive statistics (Python v3.12), and open-text responses were qualitatively assessed using inductive thematic analysis to identify recurring themes and insights. The ranking items were sorted by median rank, and when medians were similar, the lower and upper quartiles were used to determine the ranks. The findings of the questionnaires were then correlated with the scientific literature and clinical guidelines to identify gaps in research, clinical practice, or the industry offerings.

### Ethical considerations

This study surveyed clinicians and manufacturers in the diabetic footwear sector. No health-related or sensitive data was collected. Participation to the survey was voluntary, and informed consent was obtained before filling the questionnaires. Responses were anonymised prior to the analysis. Incomplete responses were treated as revoked consent and were removed prior to the analysis. Both questionnaires were designed using a web-based application compliant with General Data Protection Regulation (GDPR), and are available in the supplementary materials.

## Results

**Fig. 1:**
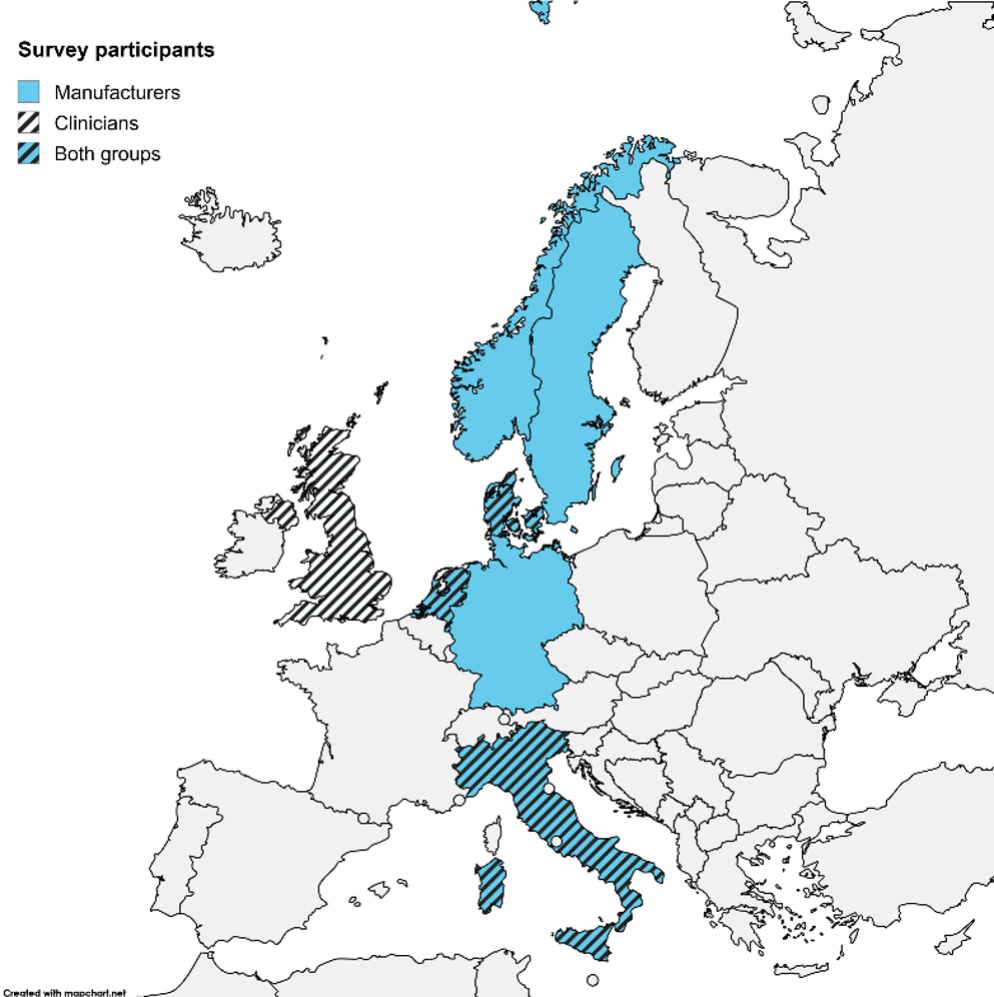
Geographical distribution of participating manufacturers and clinicians

Of the 44 invited manufacturers, 9 completed the questionnaire within the data collection timeframe (20% response rate). The second questionnaire was completed by 12 of the 16 clinicians invited (75% response rate). The following two sections report the key findings of the questionnaires (Fig. 1).

### Manufacturers’ perspective

Three out of the nine manufacturers that completed the survey were based in Italy, two in the Netherlands, one in Denmark, one in Germany, one in Norway, and one in Sweden. The manufacturers varied in size, with a median of 80 employees (range 18–350). Seven of the representatives who completed the survey had more than 10 years of experience, while two had 5–10 years in their current roles. Seven of the manufacturers specialise in off-the-shelf and custom/semi-custom shoes, with the following average shoe prices: < 100€ (*n* = 1), 100–200€ (*n* = 3), 200–300€ (*n* = 2), and > 300€ (*n* = 1). While two specialise in custom/semi-custom-made shoes, selling on average > 300€ shoes (*n* = 2). The participants indicated the targeted risk levels according to the IWGDF guidelines [5], as people at high risk (*n* = 8), medium risk (*n* = 6), low risk (*n* = 6), and very low risk (*n* = 6) for DFUs.

About half of the manufacturers (*n* = 5) reported having a research and development (R&D) department. Most respondents indicated using data-driven approaches to footwear design (*n* = 8) and incorporating data and information from scientific literature (*n* = 7). They further reported following the IWGDF guidelines (*n* = 6), the scientific literature (*n* = 1), “orthopaedic traditions” (*n* = 1), and a “state of the art design protocol for custom made footwear” (*n* = 1) in designing their diabetic shoes. When asked whether they test the effectiveness of the features of their shoes, the majority answered “Yes” (*n* = 6), while some answered “Sometimes” (*n* = 2) and one answered “No” (*n* = 1). Moreover, the majority of the respondents reported developing their own midsoles (*n* = 8), outsoles (*n* = 7), and shoe lasts (*n* = 7).

**Fig. 2:**
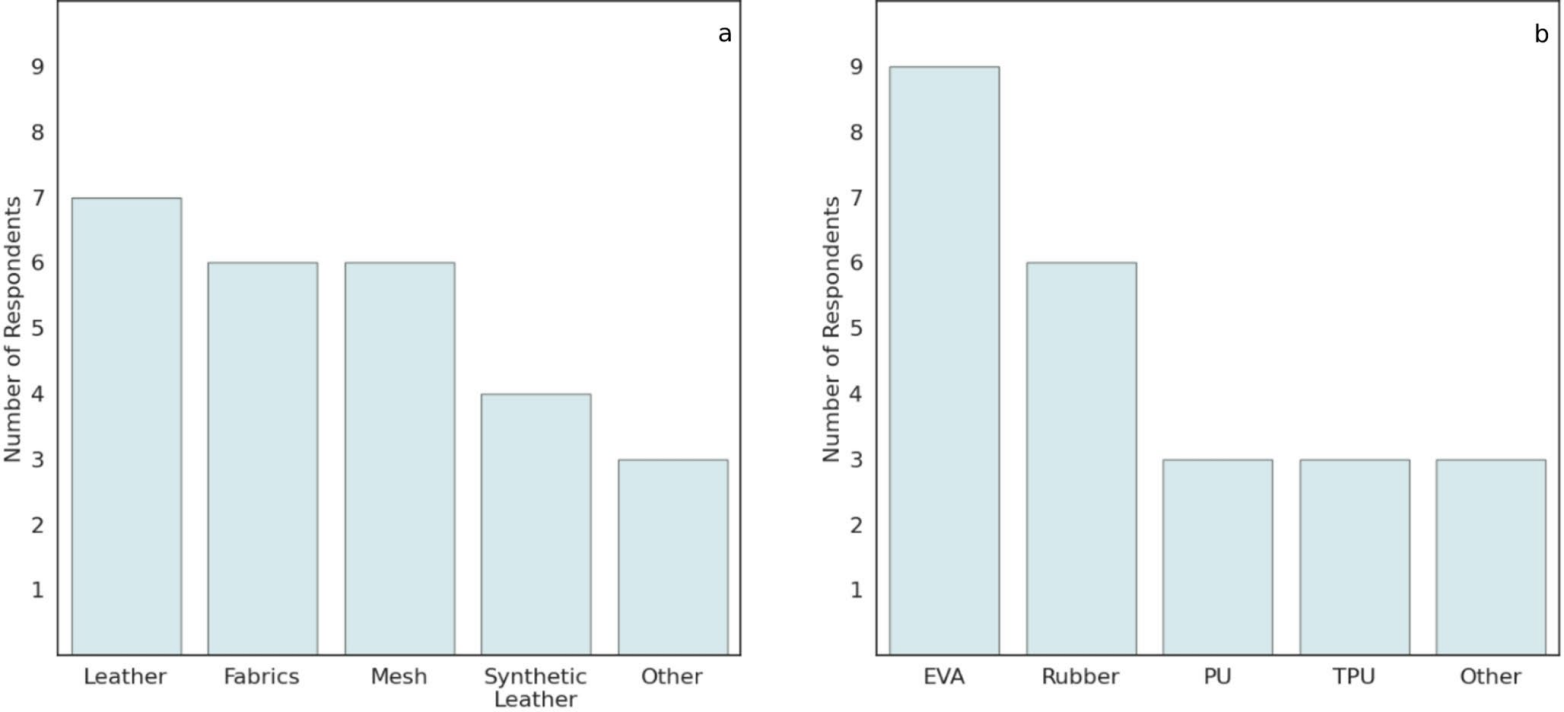
The materials used to make shoe uppers **a**, and shoe midsole/outsoles **b**, selected by the respondents in the manufacturers’ questionnaire (*n* = 9)

For the diabetic shoe upper, the respondents specified using leather (*n* = 7), fabrics (*n* = 6), mesh (*n* = 6), synthetic leather (*n* = 4), and other materials (*n* = 3), including combinations of materials or other synthetic materials. As for the shoe midsole/outsole, they reported using ethylene-vinyl acetate (EVA) (*n* = 9), rubber (*n* = 6), polyurethane (PU) (*n* = 3), thermoplastic polyurethane (TPU) (*n* = 3), or other materials (*n* = 3), for instance, a specific combination of EVA and rubber, or thermoplastic rubber (Fig. 2).

**Fig. 3:**
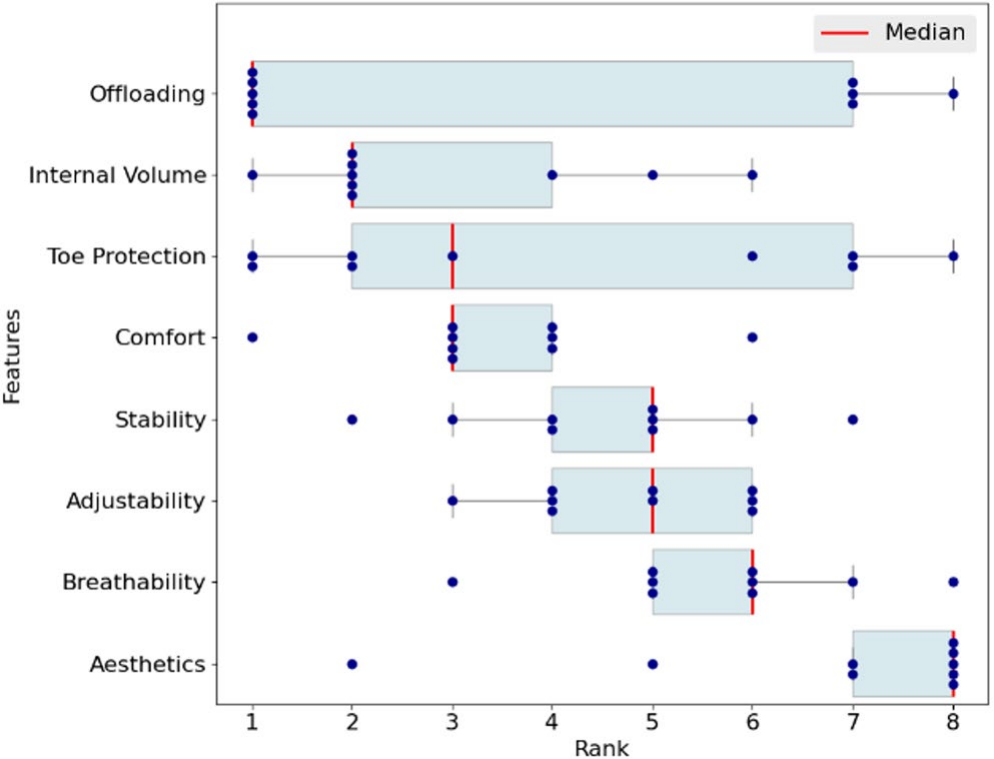
Boxplots showing the ranking (1 = most important, 8 = least important) of perceived importance for eight features of diabetic footwear among manufacturers (*n* = 9)

Respondents were asked to rank eight specific shoe features by importance, with 1 indicating the most important. The results shown in Fig. 3 highlight the significance of offloading, higher internal volume and toe protection as the top three priorities for diabetic shoes. Offloading showed the most significant variation in ranking positions. Comfort, stability, adjustability, and breathability fell within ranks 4 to 7, while shoe aesthetics consistently ranked lowest.

Manufacturers use a variety of methods to enhance the stability of diabetic footwear. The most reported were incorporating a reinforced heel counter (*n* = 7), larger outsole (*n* = 6), and an anti-slip outsole (*n* = 6), while the use of an ankle-high upper (*n* = 4), harder midsole materials (*n* = 3), and arch support (*n* = 2) were less commonly chosen. Other responses included the use of carbon inserts (*n* = 1) and integrated internal supports (*n* = 1). One respondent from a manufacturer that produces both off-the-shelf and custom/semi-custom shoes indicated that they consider midsole hardness on a case-by-case basis, with higher-risk individuals receiving harder midsoles.

Sustainability practices were reported as only life-cycle assessment (*n* = 5), a combination of life-cycle assessment and the use of vegan leathers (*n* = 2), and biodegradable materials (*n* = 1). Two respondents reported no sustainability practices or being unsure.

Five respondents reported their recent innovations, including new footwear materials, a new outsole shape, a new shoe closure system, and lowering the toe spring and heel height while including the rocker on the midsole instead of the shoe last.

Manufacturers reportedly use several methods to gather user feedback about their products. The most common approaches were through medical doctors and podiatrists (*n* = 8), as well as direct patient feedback (*n* = 7), followed by customer reviews (*n* = 6), focus groups (*n* = 5), clinical trials (*n* = 3), and surveys (*n* = 1). Finally, three respondents indicated the use of additive manufacturing techniques to produce insoles (*n* = 3), outsoles (*n* = 1), and lasts (*n* = 1). The use of Artificial Intelligence tools is reported for marketing purposes (*n* = 1), production (*n* = 1), and “in all sections” (*n* = 1).

### Clinicians’ perspective

The survey respondents were located in the UK (*n* = 6), Denmark (*n* = 3), the Netherlands (*n* = 2), and Italy (*n* = 1) and had more than 20 years (*n* = 6), 10 to 20 years (*n* = 4), and 5 to 10 years (*n* = 2) of experience working in diabetic foot care. Eight of the participants were podiatrists, three were physicians and one was a researcher in clinical biomechanics. Four participants reported that the IWGDF guidelines (*n* = 2), Dutch and IWGDF guidelines (*n* = 1), and Australian guidelines (*n* = 1) were informative for footwear recommendations. Five participants did not name any guideline they found informative, and three did not provide any answer to the question.

**Fig. 4:**
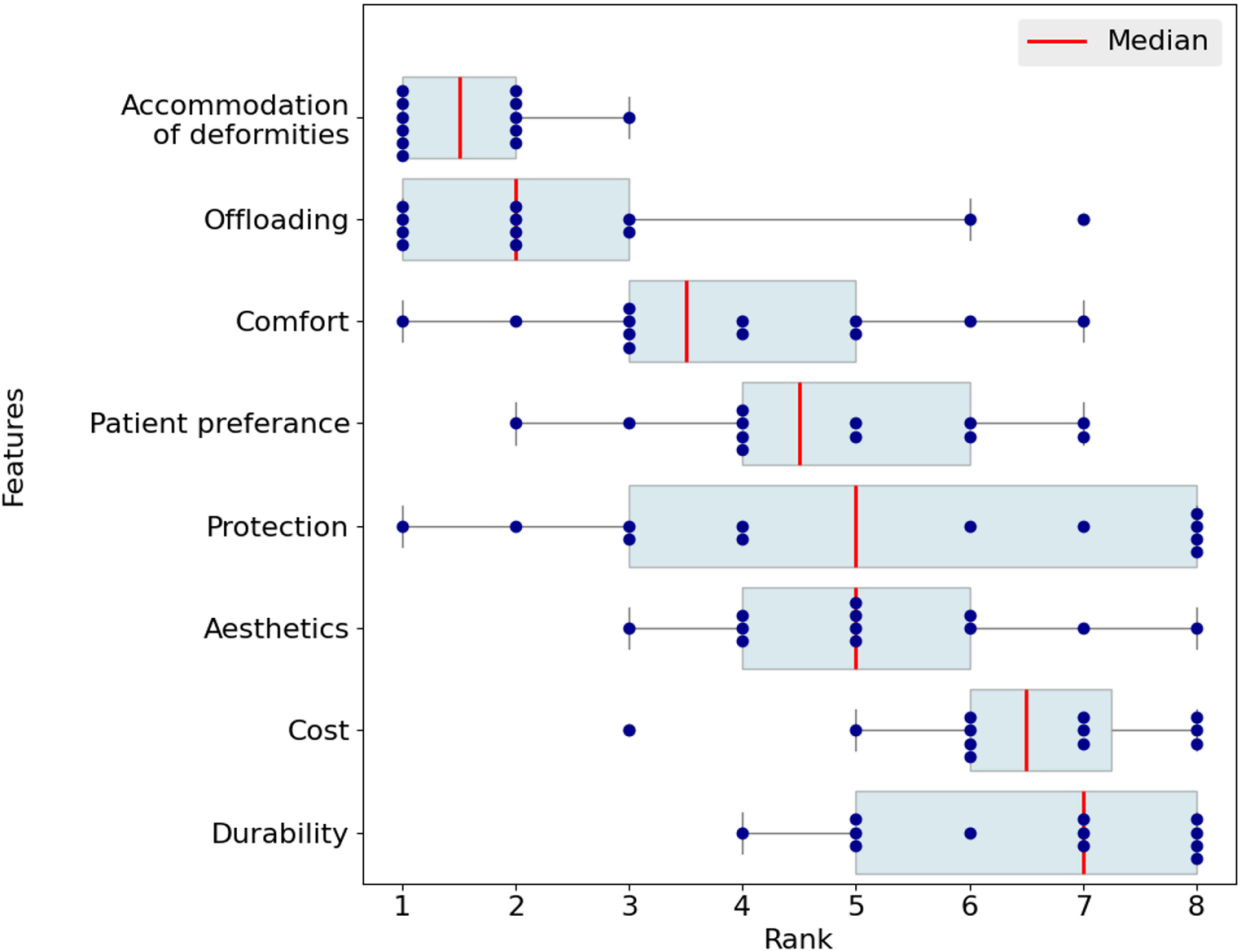
Boxplots showing the ranking (1 = most important, 8 = least important) of perceived importance of eight footwear features by clinicians when recommending shoes (*n* = 12)

The participants were asked to rank eight footwear features in order of importance for their footwear recommendations. Accommodation of foot deformities was ranked highest among the footwear features by clinicians, followed by offloading capabilities and comfort. Other factors, such as patient preference, protection, and aesthetics, were ranked in the middle, while cost and durability were the lowest-ranking features of diabetic shoes (Fig. 4).

A major gap in the industry offerings identified by clinicians was the lack of aesthetically pleasing footwear that improves adherence (*n* = 4). Other reported gaps of the industry offerings included limited accommodation of foot deformities (*n* = 3), high weight (*n* = 2), and unproven offloading properties (*n* = 1). Moreover, they identify patient-reported barriers to using diabetic shoes as aesthetics (*n* = 11), high weight (*n* = 10), cost (*n* = 5), discomfort (*n* = 4), and other factors such as being “cumbersome to put on” and “lack of flexibility for different styles/occasions”. They also highlight the main factors that would influence their prescribed footwear, which included the individual’s profession (*n* = 7), as well as their physical activity and hobbies (*n* = 4).

The final questions of the survey aimed to determine whether the clinicians needed any additional information from the shoe manufacturers or had any additional comments. The clinicians emphasised the importance of easy-to-read data leaflets that clearly communicate the specifications of the footwear, such as the dimensions of the toe box, rocker profile, stiffness properties, and materials, as well as scientific evidence on the footwear’s effectiveness.

## Discussion

This study aimed to identify current practices, needs, challenges and opportunities in diabetic footwear by integrating insights from therapeutic shoe manufacturers and clinicians working in diabetic foot care.

Although the presence of an R&D department was reported by only about half of the respondents, the survey findings indicate a trend toward a data-driven approach to footwear development, with a strong focus on integrating scientific literature into the design process. Moreover, five manufacturers reported testing the effectiveness of the footwear features. While this seems to partially address the clinicians’ reported concerns about the lack of evidence on the diabetic shoes’ properties, the scientific literature regarding the offloading properties of therapeutic footwear remains limited [4].The manufacturers reported ongoing initiatives to explore new materials with optimised properties for use in diabetic footwear. However, data on midsole and outsole materials, their mechanical properties, and relevant biomechanical effects are scarce [11]. The results of this survey suggest that EVA is the predominant material used by diabetic shoe manufacturers, likely due to its cushioning properties, durability and resistance to chemicals and UV radiation. EVA is frequently blended with other compounds (e.g., rubber) to reduce its limitations as a midsole material [22]. Thus, the properties of EVA composites vary significantly across manufacturers, particularly in parameters such as cushioning, stiffness, and energy return. It has been shown that using different midsoles with differing rigidity levels can help redistribute plantar pressure across different regions [23], highlighting the need for adjustable stiffness in diabetic shoes. This may also be achieved through changes in foam density or the integration of carbon plate inserts, without compromising comfort [24]. Furthermore, current 3D printing technologies enable the production of shoe components with complex and personalised geometrical features, facilitating the incorporation of variable stiffness and weight reduction [25]. According to this survey, 3D printing technologies are not widely used in the industry and are mainly utilised for the production of custom plantar orthotics.

People with DM at risk of foot ulceration have several critical footwear requirements, including effective pressure offloading and an optimal fit [5]. Manufacturers primarily focus on features such as offloading effectiveness, high internal volume, and toe protection, which address the core functional needs of at-risk individuals. However, aesthetics remains lower on manufacturers’ priority list (Fig. 3). As widely reported in the literature [26–28], limited attention to appearance may negatively impact user adherence. While almost all clinicians (*n* = 11) reported that their patients often cite the appearance of therapeutic footwear as a barrier to use, the clinicians themselves assigned aesthetics a comparatively low priority, ranking it 6th among footwear properties. Other factors reported by clinicians as barriers to adherence, such as excessive weight and discomfort, are consistent with previous research on patient compliance with therapeutic footwear [28]. Overall, manufacturers and clinicians demonstrated a notable similarity in their ranking of footwear features. This could reflect the influence of seeking direct feedback from clinicians, as reported by the majority of manufacturers (*n* = 7).

Individuals affected by diabetic neuropathy have reduced gait stability, which could lead to an increased risk of falling [29]. The manufacturers reportedly employ several methods to improve stability, such as larger outsoles, reinforced heel counters, and anti-slip outsoles, which are also backed by research on shoe stability [30, 31].

Most respondents indicated the inclusion of practices such as life-cycle assessment, which evaluates the environmental impact of products from raw material extraction to manufacturing and the end-of-life stage [32]. Some manufacturers reported the effort to limit the use of leather, which could enhance product sustainability, as leather is one of the largest contributors to the environmental impact of shoe production [32]. These actions by the shoe manufacturers indicate awareness regarding sustainable practices; however, the industry could still benefit from more robust research, the adoption of sustainability guidelines, and new manufacturing techniques such as 3D printing, which could help reduce waste and decrease environmental impact [19].

While this survey provides a valuable overview of current practices and approaches to diabetic shoes across Europe, the results should be critically assessed, considering some limitations. The number of participants in either questionnaire is relatively low, which, combined with the potential for participation bias towards more research-oriented organisations, may restrict the generalisability of the findings. Moreover, only manufacturers and clinicians from high-income European countries responded to the surveys; therefore, the circumstances may be significantly different in regions with fewer financial resources. In addition, differences in the reimbursement scheme across healthcare systems of the participating countries, and sometimes different policies across regions of the same country, could have affected the responses. Nonetheless, given the difficulties in engaging with industry and clinicians on a broader scale, the current study could help inform a larger, more comprehensive pan-European survey. Further research could benefit from a wider cohort of clinicians and manufacturers with more geographical diversity.

Another limitation is the self-reported nature of these questionnaires, which might introduce bias into the results. Objective assessment of the off-the-shelf and custom-made shoes, as well as innovative approaches to footwear design, could help bridge the gap between research, clinical practice and industry and increase the usability and, in turn, patient adherence. Research targeting diabetic footwear materials could also benefit from further investigation.

Interdisciplinary approaches involving engineers, fashion designers, and clinical advisors could be beneficial in further optimising diabetic footwear. Furthermore, the demand for detailed and intuitive product specifications could be a clear next step for manufacturers to meet the needs of clinical teams and prevent the prescription of inappropriate footwear [4], which could improve the outcome of prevention and treatment strategies for the diabetic population.

## Conclusions

This study explores the evolving field of diabetic footwear from the perspectives of shoe manufacturers and clinicians. While manufacturers and clinicians agree on key functional requirements such as offloading and fit, aesthetics is given lower priority by both, despite nearly all clinicians citing aesthetics as a barrier to adherence. Some manufacturers reported shifting towards a more data-driven and evidence-based approach by incorporating objective measurements and scientific literature into their new designs. This approach could result in more effective therapeutic footwear, especially if the data are clearly communicated to clinicians. The findings suggest that future development of diabetic footwear relies on closer collaboration among stakeholders, including manufacturers, clinicians, and end users. Collaborative research with a broader and more diverse group of manufacturers and clinicians could help further confirm the findings of this study.

## Supplementary Information

Below is the link to the electronic supplementary material.

Supplementary Material 1

## Data Availability

Anonymised data is available upon reasonable request.
