# Supplementary material for: Current strategies and priorities for diabetic footwear design and production: a cross-European exploratory survey of clinicians and shoemakers
Source: Acta Diabetol. 2026 Feb 18;63(5):887–95. doi: 10.1007/s00592-026-02669-6 (PMC13219091; doi:10.1007/s00592-026-02669-6)
Supplement: Supplementary file 1 — Supplementary Material 1 [file 592_2026_2669_MOESM1_ESM.docx]

**Supplementary Information**

**Title**:

Current strategies and priorities for diabetic footwear design and production: A cross-European exploratory survey of clinicians and shoemakers

**Hadi Sarlak^1,2^, Kamran Shakir^1,2,*^, Giulia Rogati^2^, Alberto Leardini^2^, Lisa Berti^1,3^, and Paolo Caravaggi^2^**

^1^ Department of Biomedical and Neuromotor Sciences, Alma Mater Studiorum - Università di Bologna, 40136 Bologna, Italy

^2^ Movement Analysis Laboratory and Functional Evaluation of Prostheses, IRCCS Istituto Ortopedico Rizzoli, Via di Barbiano 1/10, 40136 Bologna, Italy

^3^ Physical Medicine and Rehabilitation Unit, IRCCS Istituto Ortopedico Rizzoli, 40136 Bologna, Italy

**^*^**Corresponding author: Department of Biomedical and Neuromotor Sciences ALMA MATER STUDIORUM - Università di Bologna - Via Zamboni, 33 - 40126 Bologna, Italy; P.No: +39-051-6366571, E-mail address: [kamran.shakir2@unibo.it](mailto:kamran.shakir2@unibo.it) (K.S.)

# Questionnaire aimed at the manufacturers

- Your company
  (The company name will only be used for demographics and managing responses. Companies will not be named in any research papers resulting from this survey.)
  - Name of the company
  - Headquarters
  - Approximate number of employees
- Your department within the company
  - Administration
  - Marketing
  - R&D
  - Design
  - Production
  - Other (please specify)
- Years of experience in your current role
  - Less than 1 year
  - 1-5 years
  - 6-10 years
  - More than 10 years
- What types of preventive diabetic footwear does your company produce?
  - Premade/off-the-shelf shoes
  - Custom/semi-custom shoes
  - Insoles
  - Socks
  - Other (please specify)
- Which risk levels do your diabetic footwear mainly target? (PN: Peripheral Neuropathy, PAD: Peripheral Arterial Disease)
  - Very low risk (No PN, no PAD)
  - Low risk (PN or PAD)
  - Medium risk (PN+PAD or PN/PAD + foot deformity)
  - High risk (PN/PAD + ulcer history/amputation history/end-stage renal disease)
- Upper materials primarily used for your diabetic shoes (check all that apply)
  - Leather
  - Synthetic Leather
  - Fabrics
  - Mesh
  - Other (please specify)
- Midsole/outsole materials primarily used for diabetic shoes (Check all that apply)
  - Rubber
  - EVA
  - PU
  - PEBA
  - TPU
  - Other (please specify)
- How important are the following features in diabetic footwear design? (Use the arrows or drag and drop to put the items in your desired order)
  - Breathability
  - Toe protection
  - Adjustability
  - Aesthetics
  - Offloading
  - Stability
  - Comfort
  - Internal volume
- Do you design/develop the midsoles of your diabetic shoes?
  - Yes
  - No
  - Other (please specify)
- Do you design/develop the outsoles of your diabetic shoes?
  - Yes
  - No
  - Other (please specify)
- Where are your off-the-shelf diabetic shoe lasts developed?
  - Made within the company
  - In another company
  - Other (please specify)
- What thickness of insoles does your diabetic shoes allow to be inserted?
  - Less than 6 mm
  - 6 to 8 mm
  - More than 8 mm
- How do you improve the stability of your off-the-shelf diabetic footwear? (Check all that apply)
  - Larger outsole
  - Anti-slip outsole
  - Reinforced heel counter
  - Arch support
  - Hard midsole/outsole material
  - Ankle-high upper
  - Other/comments (please specify)
- What is the average price range for the off-the-shelf diabetic footwear you produce?
  - Under €100
  - €100 - €200
  - €200 - €300
  - Over €300
- Does your company utilize sustainable practices regarding the materials and production methods for footwear? (please specify)
  - Recycled Materials
  - Biodegradable Materials
  - Vegan Leathers
  - Life Cycle Assessment
  - Other (please specify)
- Does your company have a diabetic shoe R&D department?
  - No
  - Yes
  - R&D is outsourced (academic/research centers or similar)
  - Other/comments (please specify)
- Are your footwear designs data-driven? (E.g., data from pressure plate/3D scans/etc.
  - Yes
  - No
  - Outsourced
  - Other/comments (please specify)
- Do you normally test the effectiveness of the features of your shoes?
  - Yes
  - No
  - Sometimes
  - Other/comments (please specify)
- Are your products designed and optimized according to the analysis of scientific literature?
  - Yes
  - No
  - Sometimes
  - Other/comments (please specify)
- Do you follow any guidelines or standards for designing diabetic footwear?
  - International Working Group on the Diabetic Foot (IWGDF)
  - American Diabetes Association (ADA)
  - No
  - Other (please specify)
- How often do you update your designs or develop new diabetic footwear?
  - Every 6 months
  - Annually
  - Every 2-3 years
  - Other (please specify)
- What recent innovation(s) have you introduced in your diabetic footwear lineup?
- How do you gather feedback on your diabetic footwear? (Check all that apply)
  - Surveys
  - Clinical Trials
  - Focus Groups
  - Customer Reviews
  - Direct Patient Feedback
  - Medical Doctors / Podiatrists
  - Other (please specify)
- Do you have any additional comments or any suggestions regarding current challenges/opportunities in the design of diabetic footwear?

Follow-up questions

- Is your company using additive manufacturing (3d printing) to make shoe parts?
  - Midsole
  - Outsole
  - Upper
  - Insole
  - Other (please specify)
- Is your company using artificial intelligence (AI) for any of the following?
  - Designing shoe parts
  - Production
  - Marketing
  - Sales
  - Other (please specify)

# Questionnaire aimed at the clinicians

- What is your professional background?
  - Podiatrist
  - Physician
  - Orthotist/Prosthetist
  - Shoe Technician
  - Researcher/Scientist
  - Other (please specify)
- How many years of experience do you have working with diabetic foot patients?
  - < 5 years
  - 5-10 years
  - 11-20 years
  - >20 years
- In which country do you primarily practice?
- Based on your clinical experience, what is the current standard of care regarding footwear provision for patients in IWGDF Risk Group 2 (moderate risk) in your practice or region?
  - Note: The International Working Group on the Diabetic Foot Classifies the person as being "moderate risk" when one of the following conditions are met:
    - Loss of protective sensation + peripheral arterial disease
    - Loss of protective sensation + foot deformity
    - Peripheral arterial disease + foot deformity
- What types of footwear interventions do you typically recommend or provide for patients in IWGDF Risk Group 2? (Select all that apply)
  - Standard off-the-shelf diabetic shoes
  - Semi-customizable footwear
  - Custom-made footwear
  - Therapeutic insoles (prefabricated)
  - Therapeutic insoles (custom-made)
  - Other (please specify)
- What are the primary factors you consider for this patient group when recommending or providing footwear? (Please rank in order of importance)
  - Offloading capabilities
  - Accommodation of foot deformities
  - Fit and comfort
  - Protection from external hazards
  - Durability
  - Appearance/aesthetics
  - Cost
  - Patient preference
- Are there any peer-reviewed studies or guidelines that you have found most informative for moderate-risk footwear?
- Do you see any gaps in industry offerings for off-the-shelf diabetic shoes? (please specify)
- What areas of development would you prioritize? Are there any features you would like to see in off-the-shelf diabetic shoes?
- What barriers do patients cite to wearing prescribed footwear?
  - Cost
  - Aesthetics
  - Discomfort
  - Weight
  - Other (please specify)
- Does any environmental or lifestyle factor (e.g., weather, work type, functional demand) influence your prescription? (Please specify)
- What do you usually specify in your footwear prescription? (check all that apply)
  - Specific company
  - Specific product/shoe model
  - Specific properties of the shoe (e.g., stiffness, rocker profile)
  - I only mention the need for diabetic shoes
  - Other (please specify)
- What (if any) information or support do you need from footwear manufacturers to better select and provide off-the-shelf footwear for your moderate-risk patients?
- Do you have any additional comments on this topic?
